# Attention Deficit Hyperactivity Disorder Symptoms, Sleepiness and Accidental Risk in 36140 Regularly Registered Highway Drivers

**DOI:** 10.1371/journal.pone.0138004

**Published:** 2015-09-16

**Authors:** Pierre Philip, Jean-Arthur Micoulaud-Franchi, Emmanuel Lagarde, Jacques Taillard, Annick Canel, Patricia Sagaspe, Stéphanie Bioulac

**Affiliations:** 1 Services d'explorations fonctionnelles du système nerveux, Clinique du sommeil, CHU de Bordeaux, Place Amélie Raba-Léon, 33076, Bordeaux, France; 2 USR CNRS 3413 SANPSY, CHU Pellegrin, Université de Bordeaux, Bordeaux, France; 3 INSERM U897, ISPED, Equipe PPCT, Université de Bordeaux, 33000, Bordeaux, France; 4 Association des Sociétés Françaises d'Autoroutes, Paris, France; 5 Pôle Universitaire Psychiatrie Enfants et Adolescents, Centre Hospitalier Charles Perrens, 121 rue de la Béchade, 33076, Bordeaux, France; Oasi Institute for Research and Prevention of Mental Retardation, ITALY

## Abstract

**Background:**

Attention Deficit Hyperactivity Disorder (ADHD) is a frequent neurodevelopmental disorder that increases accidental risk. Recent studies show that some patients with ADHD can also suffer from excessive daytime sleepiness but there are no data assessing the role of sleepiness in road safety in patients with ADHD. We conducted an epidemiological study to explore sleep complaints, inattention and driving risks among automobile drivers.

**Methods and Findings:**

From August to September 2014, 491186 regular highway users were invited to participate in an Internet survey on driving habits. 36140 drivers answered a questionnaire exploring driving risks, sleep complaints, sleepiness at the wheel, ADHD symptoms (Adult ADHD Self-Report Scale) and distraction at the wheel. 1.7% of all drivers reported inattention-related driving accidents and 0.3% sleep-related driving accidents in the previous year. 1543 drivers (4.3%) reported ADHD symptoms and were more likely to report accidents than drivers without ADHD symptoms (adjusted OR = 1.24, [1.03–1.51], p < .021). 14.2% of drivers with ADHD symptoms reported severe excessive daytime sleepiness (Epworth Sleepiness Scale >15) versus 3.2% of drivers without ADHD symptoms and 20.5% reported severe sleepiness at the wheel versus 7.3%. Drivers with ADHD symptoms reported significantly more sleep-related (adjusted OR = 1.4, [1.21–1.60], p < .0001) and inattention-related (adjusted OR = 1.9, [1.71–2.14], p<0001) near misses than drivers without ADHD symptoms. The fraction of near-misses attributable to severe sleepiness at the wheel was 4.24% for drivers without ADHD symptoms versus 10,35% for drivers with ADHD symptoms.

**Conclusion:**

Our study shows that drivers with ADHD symptoms have more accidents and a higher level of sleepiness at the wheel than drivers without ADHD symptoms. Drivers with ADHD symptoms report more sleep-related and inattention-related near misses, thus confirming the clinical importance of exploring both attentional deficits and sleepiness at the wheel in these drivers. Road safety campaigns should be improved to better inform drivers of these accidental risks.

## Introduction

Attention Deficit Hyperactivity Disorder (ADHD) is a developmental disorder. According to the Diagnostic and Statistical Manual of Mental Disorders, 5^th^ edition (DSM-5, [1]), ADHD includes symptoms of inattention, hyperactivity and impulsivity with clear evidence of impairment in multiple domains and onset of symptoms by age twelve [1]. Longitudinal studies have documented the persistence of ADHD into adulthood, and impairing symptoms of ADHD may persist into adulthood in 50 to 65% of cases [2, 3]. The prevalence of ADHD in adults has been estimated to be 2.9% in France [4].

ADHD adults suffer from poor regulation of attention and behavior with a broad impact on life-functioning [5, 6]. ADHD has been shown to be associated with a higher risk of motor vehicle accidents and impaired driving performance [6–8]. Driving involves multiple complex cognitive functions, including perception, motor coordination and executive function, which are usually impaired in patients with ADHD [9, 10]. Several studies and meta-analyses have consistently shown that ADHD can impair driving ability. ADHD subjects cause three to four times more accidents as control subjects. They tend to drive more impulsively and less safely, cause more accidents and more traffic violations, particularly speeding, and receive more driving license suspensions and registrations by traffic authorities than control subjects [6–8, 11–14]. The association between ADHD and impaired driving performance has been attributed to ADHD core symptoms (inattention, impulsivity). For example, they are more prone to anger on the road (road rage), aggressiveness, impulsivity, risk taking while driving. Moreover, recent studies have focused on specific risky situations for ADHD subjects. Thus, ADHD adult drivers appear to be more vulnerable while driving under monotonous conditions such as on highways [15, 16]. This may be due to the fact that ADHD drivers could be more prone to mind-wandering, which has been recently demonstrated to be associated with a higher risk of crashes [17]. Another not exclusive explanation could be related to the fact that ADHD patients report excessive daytime sleepiness [18] and suffer from sleep disorders [19–21].

Indeed, excessive daytime sleepiness is primary cause of traffic accidents on highways [22–25] and accounts for up to 20% of total traffic accidents [22, 26–29]. Sleepiness at the wheel is responsible from near misses which highly predict driving accidents. Drivers being forced to stop because of excessive sleepiness at the wheel are also at risk for future sleep related accidents [24, 30].

While sleepiness and inattention at the wheel are two very different symptoms usually concerning different type of patients, recent work from our team on a very small sample of patients [18] showed that a significant proportion of ADHD patients present a high objective level of sleepiness as measured by the Maintenance of Wakefulness test. This finding also found in children with ADHD [31, 32] questions the physiopathology of ADHD and the behavioral risk related to this disease.

Interestingly, all studies to date looking at the cognitive handicap of these patients have neglected the alertness dimension of this disorder, mainly because ADHD patients were considered to present cognitive impairments related to attention disorders associated to dopamine deficits. Methylphenidate, which regulates dopamine activity, reduces the risk of accidents in ADHD patients [33] and significantly reduces excessive daytime sleepiness [34]. The state of many ADHD patients could thus be improved through an "alerting" action and not simply an "attention" improvement effect.

To further explore these complex relationships, we collaborated with the French highway association (AFSA) on a new Internet epidemiological survey of a large sample of highway users. The main objective of this study was to explore the links between ADHD symptoms, sleepiness and accidental risk in a population of regular French highway users.

## Methods

### 1.1. Participants

With the assistance of the French highway association (AFSA, Association Française des Sociétes d’Autoroutes), we gained access to a database of registered drivers who used a computerized pay system to avoid long queues at highway toll booths.

### 1.2.Procedure

The Fondation AFSA conducts regular surveys via the Internet to improve their knowledge and understanding of drivers’ needs and behavior. An email newsletter was sent to AFSA highway Internet subscribers describing the main scientific objectives of the study. A link to the study questionnaire to be completed was inserted in the newsletter.

For the survey, we used a methodology similar to that published by Powell et al. (2007) [30] and our team [24].

From August to September 2014, 491186 subscribers of the 4 major highway networks covering Metropolitan France received a newsletter inviting them to participate in an internet survey on driving habits and driving risks. 36154 subjects answered the questionnaire. 14 questionnaires were eliminated because there contained aberrant responses. The analyses were conducted on a sample of 36140 drivers. The database was anonymous in accordance with the recommendations of the “Commissions Nationale Informatique et Libertés” which ensures the ethical usage of data collected for scientific purposes.

### 1.3.Questionnaire

Our team prepared a specific Internet questionnaire for the purpose of the study. It took a mean of 15 min to be completed. Volunteers had to respond to the following:

1. Questions concerning socio-demographical characteristics (gender, age, marital status, socio-professional categories).
2. The Epworth Sleepiness Scale (ESS [35]) to explore behavioral excessive daytime sleepiness. The ESS is an eight-item self-questionnaire that asks the subject to rate his or her probability of falling asleep on a scale of increasing probability from 0 to 3 for eight different situations that most people engage in during their daily lives. 0 corresponds to no chance of falling asleep, 1 a little chance, 2 a moderate chance and 3 a very high chance. The answers for the eight questions are added together to obtain a single score. A score in the 0–8 range is considered to be normal, while a score above 15 indicates severe excessive daytime sleepiness. Severe sleepiness is correlated with the risk of traffic accidents [24, 36].
3. The ASRS-1.1 (Adult ADHD Self-Report Scale) to explore ADHD symptoms [37]. Six of the eighteen questions of the ASRS were used (part A on the ASRS) because it was found to be the most predictive of symptoms consistent with ADHD. The specificity of part A on the ASRS is 99.5% and the sensibility is 68.7% [38]. Each question can be answered on a five-item Likert scale based on a scale of frequency ranging from "Never" to "Very Often". Answers are scored as either positive or negative and the threshold is different for individual questions. Answers of "Never" and "Rarely" are always scored negative, answers of "Often and Very Often" are always scored positive, and answers of "Sometimes" are scored positively in only three of the six questions. Four or more positive answers in Part A are indicative of ADHD symptoms.
4. The Hospital Anxiety and Depression Scale (HADS) to explore anxiety and depression symptoms. The HADS is a fourteen-item scale in which seven of the items relate to anxiety and seven relate to depression. Scores for each subscale (anxiety and depression) range from 0 to 21 with scores categorized as follows: normal 0–7, mild 8–10, moderate 11–14, and severe 15–21 [39].
5. Questions concerning sleep disorders (obstructive sleep apnea syndrome (OSAS), restless legs syndrome (RLS), insomnia, narcolepsy ⁄ hypersomnia) and sleep hygiene. Patients had to respond by “yes” or “no” if they suffered from a sleep disorder, and if “yes”, they had to respond from which sleep disorder by selecting in a list of sleep disorders (obstructive sleep apnea syndrome (OSAS), restless legs syndrome (RLS), insomnia, narcolepsy ⁄ hypersomnia).
6. Questions concerning driving habits (year of obtaining license, kilometers driven per year, professional drivers, severe sleepiness at the wheel needing to stop, inattention at the wheel).
7. Questions concerning accidental risks. Near-miss driving accidents were defined as an event that had not caused any harm and therefore had limited immediate impact (e.g. inappropriate line-crossing). Driving accidents were defined as an event that had caused material damage or physical injury. Two types of accidents and near-miss driving accidents were distinguished: related to sleepiness and related to inattention [24].

### 1.4.Statistical analyses

Data were expressed as a proportion or as mean and standard deviation. We defined two groups of drivers: drivers without ADHD symptoms (part-A ASRS, subscore < 4) and drivers with ADHD symptoms (Part-A ASRS subscore ≥ 4).

First, univariate analyses with chi-squared tests on categorical variables and t-tests on continuous variables were conducted to compare the characteristics of the sample in terms of ADHD symptoms (absence or presence). Socio-demographical and clinical variables and driving habits were compared between the two groups.

Then, multivariate analyses with logistic regression were conducted to control for potential confounding effects on the relationship between ADHD symptoms (absence or presence) and: i) behavioral sleepiness variables (severe excessive daytime sleepiness on the ESS and severe sleepiness at the wheel), ii) accident variables (accident, sleepy accident, inattention accident, near-miss accident, sleepy near-miss accident, inattention near-miss accident). Socio-demographical, driving and clinical variables that were significantly different between the two groups were included in the models. The clinical variables that were included in the multivariate analysis were: pathological sleepiness (Epworth ≥ 16), sleep disorders, pathological anxiety, pathological depression and drug consumption. Statistical test of the regression analyses permitted to estimate adjusted odd ratio (OR) and its 95% confident interval were presented to show the association.

Finally, attributable fractions of severe sleepiness at the wheel needing to stop at least once per month for near-misses were estimated from the adjusted OR estimates and the prevalence of exposure in drivers with ADHD symptoms and drivers without ADHD symptoms [40].

P-values were considered significant when p<0.05. The data were analyzed using the SPSS software package, version 20.0.

## Results

### 1.5.Participants

#### 1.5.1.Population

36140 divers participated in the study. The mean age of the drivers was 55.2 years (±13.1) and 70.6% were male, 80.4% were married and 60.4% were currently employed, 4.8% were nocturnal or shift workers, and 17.8% were professional drivers. The mean score on the ESS was 7.8 (±4), the prevalence of severe excessive daytime sleepiness in the population was 3.7% (ESS>15), the prevalence of pathological anxiety was 6.2% (HADS anxiety > 10), the prevalence of pathological depression was 1.8% (HADS depression > 10), the prevalence of more than 3 glasses of alcohol per day was 6.9%, and the prevalence of a declared sleep disorder was 13.5%. The prevalence of ADHD symptoms was 4.3% (1543).

#### 1.5.2.Driving habits

Nearly half of the sample (46.1%) declared they experienced at least one episode of severe sleepiness at the wheel (i.e. requiring to stop driving), less than once a month and nearly 8% of the sample declared severe sleepiness at the wheel at least once a month. 40% of the drivers declared using their phone to call, 12.2% to write or read text messages while driving, and 41% using a GPS while driving.

#### 1.5.3.Near-miss accidents and accidents

31.1% of the drivers reported at least one near-miss accident during the previous year, 23% of the drivers reported inattention-related near-miss accidents and 10.1% sleep-related near-miss accidents. 5.7% reported at least one driving accident in the previous year. 1.7% of the drivers reported inattention-related driving accidents (n = 598) and 0.3% (n = 125) sleep-related driving accidents.

#### 1.6.Comparison of drivers with and without ADHD symptoms

The population was divided into two groups: drivers without ADHD symptoms and drivers with ADHD symptoms. Results and p-values of univariate analyses are shown in Table 1. Socio-demographic, driving, accident and clinical data were significantly different between the two groups of drivers except for the number of subjects with less than two years of holding a license and the prevalence of more than 3 glasses of alcohol per day.

**Table 1:** Demographic characteristics of 34597 drivers without ADHD symptoms versus 1543 drivers with ADHD symptoms.

Characteristics	Drivers without ADHD symptoms N = 34597 (95.7%)	Drivers with ADHD symptoms N = 1543 (4.3%)	Statistics (Khi^2^ or t-test) P value
**Socio-demographical characteristics**			
Age (years) %			
18–30	3.8%	10.2%	
31–50	30.7%	50.4%	
51–65	40.4%	29.9%	
>65	25.1%	9.5%	<0.001
Female (%)	29.3%	32.5%	0.006
Marital status (%)			
Married	80.6%	76%	
Single	7.2%	12.7%	
Separated or divorced	12.2%	11.3%	<0.001
Socio-professional categories (%)			
Current employees	59.5%	80.4%	
Retired	37.5%	15.6%	
Not working	3.0%	4.0%	<0.001
Work (%)			
Diurnal work	93.4%	95.3%	
Nocturnal work	1.0%	1.7%	
Shift work	3.7%	4.9%	0.002
**Clinical characteristics**			
Epworth (Mean, SD)	7.6 (3.9)	10.9 (4.1)	<0.001
Epworth ≥ 16	3.2%	14.2%	<0.001
Alcohol consumption > 3 glasses per day	6.9%	6.8%	0.91
Drug consumption	1.6%	3.6%	<0.001
Mean HADS anxiety	5.0 (2.9)	8.9 (3.7)	<0.001
Pathological anxiety	5.1%	30.8%	<0.001
Mean HADS depression	3.1 (2.5)	5.7 (3.4)	<0.001
Pathological depression	1.4%	9.7%	<0.001
Sleep disorder	12.9%	25.8%	<0.001
OSAS	3.9%	6.9%	<0.001
Restless leg syndrome	1%	2.9%	<0.001
Insomnia	8.8%	18.9%	<0.001
Narcolepsy-hypersomnia	0.1%	0.9%	<0.001
**Driving characteristics**			
License (years)			
Young drivers (<2 years holding license)	3.1%	2.5%	0.076
Kilometers driven per year			
0–5000	5.3%	5.8%	
5000–25000	61.7%	56.2%	
>25000	33%	38%	<0.001
Professional driver	17.7%	19.7%	0.043
Severe sleepiness at wheel needing driver to stop			
Never	47.1%	23.2%	
Less than once a month	45.6%	56.3%	
At least once a month	7.3%	20.5%	<0.001
Distraction at wheel			
Phone	39.2%	59.8%	<0.001
Text message	11.4%	28.5%	<0.001
GPS	40.1%	61.2%	<0.001

Drivers with ADHD symptoms were more female (32.5% vs 29.3%, p = .006), younger, less married (11.3% vs 12.2% p < .001), more shift workers (4.9% vs 3.7, p = .002), more professional drivers (19.7% vs 17.7%, p = .043) and drove more than 25000 km per year (38% vs 33%, p < .001). They reported more severe sleepiness at the wheel (20.5% vs 7.3%, p < .001), more frequently using their phone to call (59.8% vs 39.2%, p < .001), writing or reading text messages (28.5% vs 11.4%, p < .001) during driving and using a GPS (61.2% vs 40.1%, p < .001) than drivers without ADHD symptoms. Drivers with ADHD symptoms exhibited significantly more excessive sleepiness (ESS>15) than those without ADHD symptoms (14.2% vs 3.2%, p < .001). They also declared more psychostimulant consumption (1% vs 0.1%) and more pathological anxiety (30.8% vs 5.1% p < .001), depression (9.7% vs 1.4% p < .001) and sleep disorders (25.8% vs 12.9% p < .001).

### 1.7.Adjusted comparisons between drivers with and without ADHD symptoms

#### 1.7.1.Behavioral sleepiness

14.2% of drivers with ADHD symptoms reported severe excessive daytime sleepiness at the ESS (>15) versus 3.2% of drivers without ADHD symptoms (p < .0001). The multivariate analysis with logistic regression found that the difference remained significant after taking into account confounding factors (socio-demographical, driving and clinical variables from Table 1), (adjusted OR 3.01, [2.54–3.57], p < .0001). 20.5% of drivers with ADHD symptoms reported severe sleepiness at the wheel that required them to stop at least once per month versus 7.3% of drivers without ADHD symptoms (p < .0001). The multivariate analysis with logistic regression found that the difference remained significant after taking into account confounding factors, (adjusted OR 2.27, [2.0–2.58], p < .0001).

#### 1.7.2.Near-miss accidents

**Fig 1:**
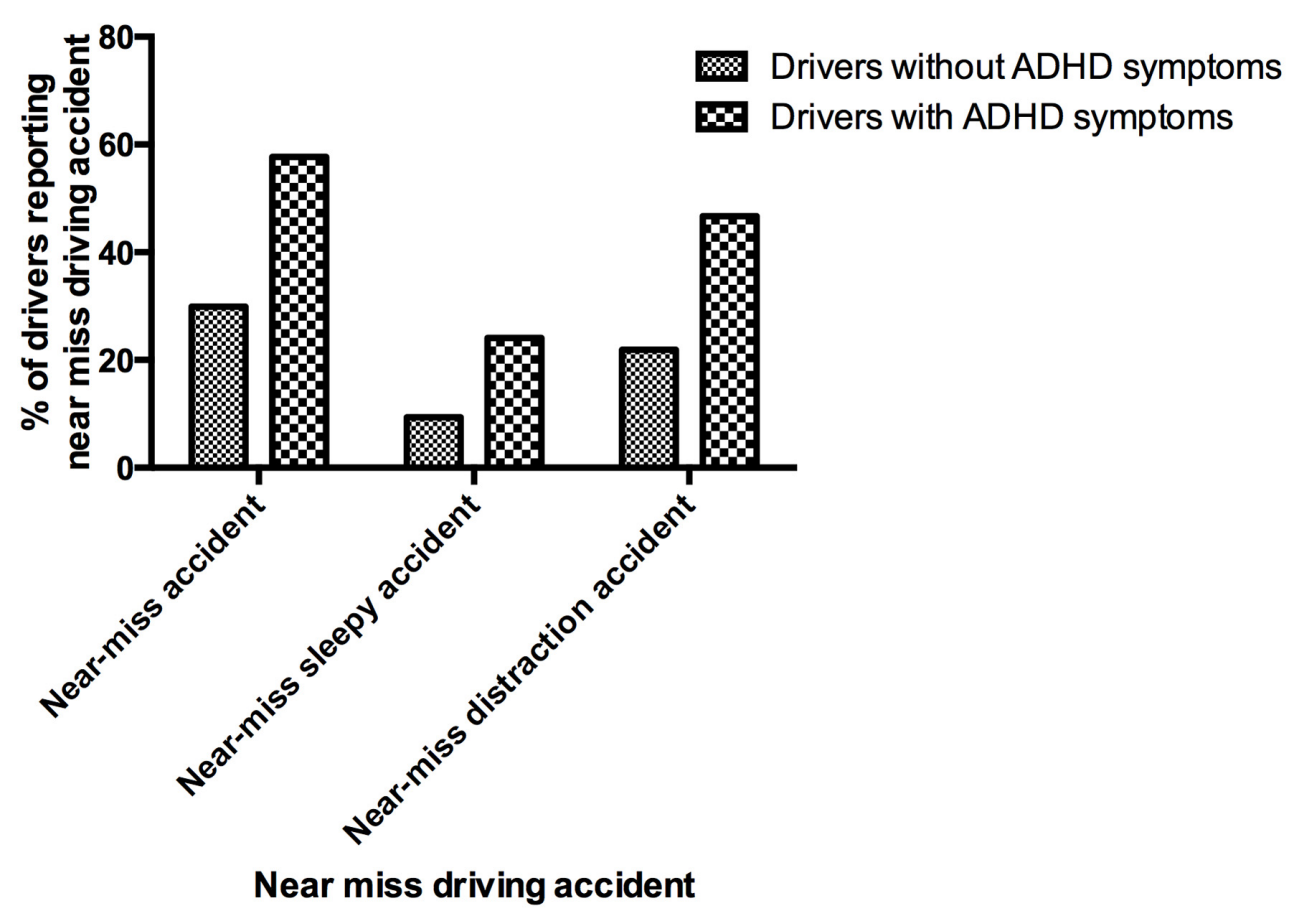
Percentages of drivers without ADHD symptoms and drivers with ADHD symptoms reporting near miss driving accidents.

57.7% of drivers with ADHD symptoms reported a near-miss accident during the previous year versus 30% of drivers without ADHD symptoms (p < .0001) (Fig 1). The multivariate analysis with logistic regression found that the difference remained significant after taking into account confounding factors (socio-demographical, driving and clinical variables from Table 1), (adjusted OR 1.84, [1.65–2.06], p < .0001). Note that pathological sleepiness (Epworth ≥ 16) (adjusted OR 1.39, [1.23–1.57]), sleep disorders (adjusted OR 1.10, [1.03–1.18]), pathological anxiety (adjusted OR 1.35, [1.22–1.48]), were also independent contributor factors for near-miss accident. Pathological depression was not contributor factor for near-miss accident.

Drivers with ADHD symptoms declared significantly more sleepiness- (adjusted OR = 1.4, [1.21–1.60], p < .0001) and inattention-related (adjusted OR = 1.9, [1.71–2.14], p<0001) near misses than drivers without ADHD symptoms. The attributable fraction to severe sleepiness at the wheel for near-misses was 10.35% [7.1–12.8] for drivers with ADHD symptoms vs 4.24% [3.9–4.5] for drivers without ADHD symptoms.

#### 1.7.3.Accidents

**Fig 2:**
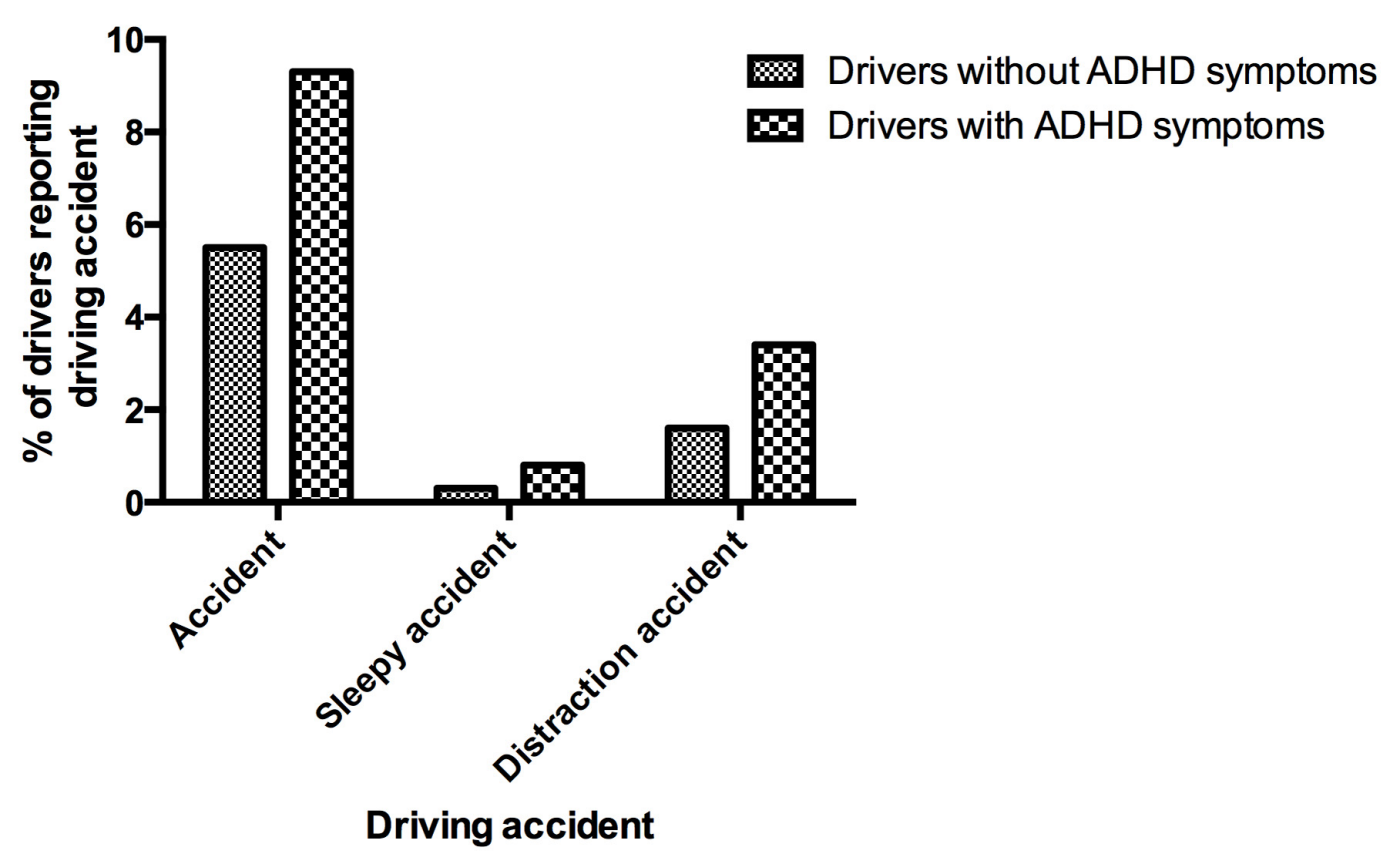
Percentages of drivers without ADHD symptoms and drivers with ADHD symptoms reporting driving accidents.

9.3% of drivers with ADHD symptoms reported at least one driving accident in previous year versus 5.5% of drivers without ADHD symptoms (p < .0001) (Fig 2). The multivariate analysis with logistic regression found that the difference remained significant after taking into account confounding factors (socio-demographical, driving and clinical variables in Table 1), adjusted OR = 1.24, [1.03–1.51], (p < .021). Drivers with ADHD symptoms declared significantly more sleepiness- and inattention-related driving accidents (adjusted OR = 1.45, [1.07–1.95], p < .015) than those without ADHD symptoms. Sleep-related accidents were significantly more numerous in drivers with ADHD symptoms than in those without ADHD symptoms in the univariate analysis but they did not remain significant after adjusting for confounding factors with the multivariate analysis.

## Discussion

This study is the first to demonstrate the higher prevalence of sleepiness at the wheel in drivers reporting ADHD symptoms than in those without ADHD symptoms. Drivers reporting ADHD symptoms also presented higher scores on the Epworth Sleepiness scale compared to those without ADHD symptoms. One possible explanation is that this population reports a higher percentage of nocturnal sleep disorders (i.e. obstructive sleep apnea syndrome, restless leg syndrome, insomnia). Another explanation, as demonstrated in one of our previous publications, is that ADHD patients suffer from objective pathological sleepiness independently of nocturnal sleep disorders [18]. This goes in line with the fact that excessive daytime sleepiness in the present study remained significantly different between the two groups after adjusting for nocturnal sleep disorders. Because excessive daytime sleepiness is associated with cognitive impairment [41, 42] which is frequently reported in ADHD patients, our results raise questions about the impact of sleepiness and attentional disorders in the daily life functioning of patients.

ADHD patients are known to exhibit an increased risk of driving accidents owing to lack of attention and subsequent cognitive impairment [8]. ADHD symptoms negatively affect driving performance and several studies have reported a higher risk of driving accidents in untreated ADHD patients. Our study clearly confirms the higher prevalence of near misses and accidents in drivers with AHDH symptoms compared to those without ADHD symptoms. Interestingly, drivers with ADHD symptoms declared more inattention near-miss driving accidents but also more sleep-related near misses.

Sleepiness is a well-known cause of accidents owing to behavioral reasons (i.e. sleep deprivation) [24, 43] and medical condition (i.e. obstructive sleep apnea, narcolepsy-hypersomnia) [44–46]. Up to 20% of car accidents can be attributed to sleepiness at the wheel and this symptom remains the primary cause of death on French highways [22, 25–29]. Our findings combined with those of previous studies tend to show that the vulnerability to accidents in ADHD cannot be only explained solely by lack of attention and subsequent cognitive impairment but could also be due to the dysregulation of waking systems. The interaction of attentional deficit with dysregulation of waking system on the accidental risk has to be explored in order to better understand the negative impact of inattention versus sleepiness in the adults with ADHD. From a neurobiological perspective, waking systems (i.e. orexin, histamin) which promote arousal and maintain wakefulness [47] could play a role in the pathophysiology of ADHD. Indeed, our recent work in a small sample of patients showed that a large proportion of ADHD adults present objective excessive daytime sleepiness which confirm that altered level of alertness can be found in adult ADHD patients [18]. These recent results confirm findings observed in children and corroborate the hypothesis of alterations in the hypocretin/orexin neurotransmitter system in this disorder [48]. Moreover, stimulant medications are often efficient in reducing ADHD symptomatology [5, 49, 50]. Several studies using on-road driving observations and driving simulator tests have shown that stimulant treatment improves driving ability in adult ADHD [51–59]. Another very recent study confirms the high risk of accidents in these patients and shows the positive impact of methylphenidate on the occurrence of traumatic events [33]. Thus, we hypothesize that some ADHD patients with sleepiness could be improved by the “alerting” effect of stimulants.

This study has several limitations. First, because we used an internet survey we got a much lower rate of response than can be obtained when direct telephone interviews are performed. Since the aim of the study was not to establish prevalence data but rather an association between symptoms and driving risk, we believe that this method of selection does not affect the validity of our results. Second, the groups were defined on the basis of ADHD symptoms and not on an ADHD diagnosis based on the DSM criteria and a clinical interview. However, ADHD symptoms were frequently (4.3%) observed in an active population (i.e. drivers), which is in line with the estimated ADHD prevalence of 2.9% in France [4]. Moreover, the presence of ADHD symptoms based on the ASRS is strongly correlated to the presence of ADHD diagnosis [37, 38]. Indeed, the six questions ASRS screener used in the present study, present a very high specificity (99.5%) suggesting that the test rarely gives positive results in non-ADHD patients [38]. However, anxiety symptoms and worrying could mimic ADHD symptoms, and the result of the present study need to be confirmed with a population of ADHD patients diagnosed with a standardized face-to-face clinical interview performed by an experimented clinicians according DSM-5 ADHD diagnostic criteria. Third, we did not find a significant difference between drivers with ADHD symptoms and drivers without ADHD symptoms with regard to sleepy accidents after adjusting for confounding factors (socio-demographical, driving and clinical variables). However, despite the very high number of drivers included in the study, only 125 reported sleepiness-related driving accidents and only 12 drivers with ADHD symptoms reported them. Thus, a lack of power may explain the fact that the results were no longer significant in the multivariate analysis. Fourth, sleep disorders were assessed on the basis of drivers’ testimonials but there was no clinical interview to confirm the disease or treatment. Nevertheless, our sample reflects the prevalence of psychiatric and sleep disorders in epidemiological studies performed in western populations [60–65].

Despite these limitations, the present study confirms the clinical importance of exploring both attentional deficits and sleepiness in subjects who complain of ADHD symptoms, in particular regarding the risk of driving accidents. When they receive an ADHD diagnosis, patients should be made aware of the risk that their propensity for distractive behavior and sleepiness may have on their driving. The present study also confirms the neurophysiological interest of studying the link between alertness and cognition in ADHD in line with the model that involves a deficit in alertness in the pathophysiology of ADHD [66–68]. Alerting drugs could be a promising therapeutic approach to reducing the risk involved when ADHD patients concerned by sleepiness take the wheel.

## References

[1] American Psychiatric Association. Diagnostic and statistical manual of mental disorders 5thed. ed. Washington, DC: American Psychiatric Association; 2013.

[2] Faraone SV, Biederman J, Mick E. The age-dependent decline of attention deficit hyperactivity disorder: a meta-analysis of follow-up studies. Psychol Med. 2006;36(2):159–65. Epub 2006/01/20. S003329170500471X [pii] 10.1017/S003329170500471X . 16420712

[3] Lara C, Fayyad J, de Graaf R, Kessler RC, Aguilar-Gaxiola S, Angermeyer M, et al Childhood predictors of adult attention-deficit/hyperactivity disorder: results from the World Health Organization World Mental Health Survey Initiative. Biol Psychiatry. 2009;65(1):46–54. Epub 2008/11/14. 10.1016/j.biopsych.2008.10.005S0006-3223(08)01203-1 [pii]. 19006789 PMC2629074

[4] Caci HM, Morin AJ, Tran A. Prevalence and correlates of attention deficit hyperactivity disorder in adults from a French community sample. J Nerv Ment Dis. 2014;202(4):324–32. Epub 2014/03/22. 10.1097/NMD.0000000000000126 . 24647218

[5] Biederman J, Faraone SV, Spencer TJ, Mick E, Monuteaux MC, Aleardi M. Functional impairments in adults with self-reports of diagnosed ADHD: A controlled study of 1001 adults in the community. J Clin Psychiatry. 2006;67(4):524–40. Epub 2006/05/04. . 16669717 10.4088/jcp.v67n0403

[6] Barkley RA, Murphy KR, Dupaul GI, Bush T. Driving in young adults with attention deficit hyperactivity disorder: knowledge, performance, adverse outcomes, and the role of executive functioning. J Int Neuropsychol Soc. 2002;8(5):655–72. Epub 2002/08/08. . 12164675 10.1017/s1355617702801345

[7] Fischer M, Barkley RA, Smallish L, Fletcher K. Hyperactive children as young adults: driving abilities, safe driving behavior, and adverse driving outcomes. Accid Anal Prev. 2007;39(1):94–105. Epub 2006/08/22. S0001-4575(06)00113-8 [pii] 10.1016/j.aap.2006.06.008 . 16919226

[8] Jerome L, Segal A, Habinski L. What we know about ADHD and driving risk: a literature review, meta-analysis and critique. J Can Acad Child Adolesc Psychiatry. 2006;15(3):105–25. Epub 2008/04/09. 18392181 PMC2277254

[9] Barkley RA, editor. Attention Deficit Hyperactivity Disorder: A Handbook for Diagnosis and Treatment, 3rd ed. New York: Guilford; 2005.

[10] Barkley RA. Driving risks in adults with ADHD: yet more evidence and a personal story. ADHD Rep. 2006;14(5):1–9.

[11] Barkley RA, Guevremont DC, Anastopoulos AD, DuPaul GJ, Shelton TL. Driving-related risks and outcomes of attention deficit hyperactivity disorder in adolescents and young adults: a 3- to 5-year follow-up survey. Pediatrics. 1993;92(2):212–8. Epub 1993/08/01. . 8337019

[12] Nada-Raja S, Langley JD, McGee R, Williams SM, Begg DJ, Reeder AI. Inattentive and hyperactive behaviors and driving offenses in adolescence. J Am Acad Child Adolesc Psychiatry. 1997;36(4):515–22. Epub 1997/04/01. S0890-8567(09)62522-3 [pii] 10.1097/00004583-199704000-00014 . 9100426

[13] Woods SP, Lovejoy DW, Stutts ML, Ball JD, Fals-Stewart W. Comparative efficiency of a discrepancy analysis for the classification of Attention-Deficit/Hyperactivity Disorder in adults. Arch Clin Neuropsychol. 2002;17(4):351–69. Epub 2003/11/01. S0887617701001202 [pii]. . 14589720

[14] Woodward LJ, Fergusson DM, Horwood LJ. Driving outcomes of young people with attentional difficulties in adolescence. J Am Acad Child Adolesc Psychiatry. 2000;39(5):627–34. Epub 2000/05/10. S0890-8567(09)66223-7 [pii] 10.1097/00004583-200005000-00017 . 10802981

[15] Reimer B, D'Ambrosio LA, Coughlin JF, Fried R, Biederman J. Task-induced fatigue and collisions in adult drivers with attention deficit hyperactivity disorder. Traffic Inj Prev. 2007;8(3):290–9. Epub 2007/08/22. 781416026 [pii] 10.1080/15389580701257842 . 17710720

[16] Reimer B, Mehler B, D'Ambrosio LA, Fried R. The impact of distractions on young adult drivers with attention deficit hyperactivity disorder (ADHD). Accid Anal Prev. 2010;42(3):842–51. Epub 2010/04/13. 10.1016/j.aap.2009.06.021S0001-4575(09)00154-7 [pii]. . 20380911

[17] Galera C, Orriols L, M'Bailara K, Laborey M, Contrand B, Ribereau-Gayon R, et al Mind wandering and driving: responsibility case-control study. Bmj. 2012;345:e8105 Epub 2012/12/18. 10.1136/bmj.e8105 ; PubMed Central PMCID: PMCPmc3521876. 23241270 PMC3521876

[18] Bioulac S, Chaufton C, Taillard J, Claret A, Sagaspe P, Fabrigoule C, et al Excessive daytime sleepiness in adult patients with ADHD as measured by the Maintenance of Wakefulness Test, an electrophysiologic measure. J Clin Psychiatry. 2015 Epub 2015/01/23. 10.4088/JCP.14m09087 . 25610980

[19] Hvolby A. Associations of sleep disturbance with ADHD: implications for treatment. Atten Defic Hyperact Disord. 2015;7(1):1–18. Epub 2014/08/17. 10.1007/s12402-014-0151-0 25127644 PMC4340974

[20] Yoon SY, Jain U, Shapiro C. Sleep in attention-deficit/hyperactivity disorder in children and adults: past, present, and future. Sleep Med Rev. 2012;16(4):371–88. Epub 2011/10/29. 10.1016/j.smrv.2011.07.001S1087-0792(11)00071-2 [pii]. . 22033171

[21] Cortese S, Brown TE, Corkum P, Gruber R, O'Brien LM, Stein M, et al Assessment and management of sleep problems in youths with attention-deficit/hyperactivity disorder. J Am Acad Child Adolesc Psychiatry. 2013;52(8):784–96. Epub 2013/07/25. 10.1016/j.jaac.2013.06.001S0890-8567(13)00374-2 [pii]. . 23880489

[22] Horne JA, Reyner LA. Sleep related vehicle accidents. Bmj. 1995;310(6979):565–7. Epub 1995/03/04. ; PubMed Central PMCID: PMCPmc2548939. 7888930 10.1136/bmj.310.6979.565 PMC2548939

[23] Pack AI, Pack AM, Rodgman E, Cucchiara A, Dinges DF, Schwab CW. Characteristics of crashes attributed to the driver having fallen asleep. Accid Anal Prev. 1995;27(6):769–75. Epub 1995/12/01. . 8749280 10.1016/0001-4575(95)00034-8

[24] Philip P, Sagaspe P, Lagarde E, Leger D, Ohayon MM, Bioulac B, et al Sleep disorders and accidental risk in a large group of regular registered highway drivers. Sleep Med. 2010;11(10):973–9. Epub 2010/10/22. 10.1016/j.sleep.2010.07.010 . 20961809

[25] Sagaspe P, Taillard J, Bayon V, Lagarde E, Moore N, Boussuge J, et al Sleepiness, near-misses and driving accidents among a representative population of French drivers. J Sleep Res. 2010;19(4):578–84. Epub 2010/04/23. 10.1111/j.1365-2869.2009.00818.x . 20408921

[26] Philip P, Vervialle F, Le Breton P, Taillard J, Horne JA. Fatigue, alcohol, and serious road crashes in France: factorial study of national data. BMJ. 2001;322(7290):829–30. Epub 2001/04/06. 11290636 10.1136/bmj.322.7290.829 PMC30559

[27] Connor J, Whitlock G, Norton R, Jackson R. The role of driver sleepiness in car crashes: a systematic review of epidemiological studies. Accid Anal Prev. 2001;33(1):31–41. Epub 2001/02/24. S0001-4575(00)00013-0 [pii]. . 11189120 10.1016/s0001-4575(00)00013-0

[28] Connor J, Norton R, Ameratunga S, Robinson E, Civil I, Dunn R, et al Driver sleepiness and risk of serious injury to car occupants: population based case control study. Bmj. 2002;324(7346):1125 Epub 2002/05/11. ; PubMed Central PMCID: PMCPmc107904. 12003884 10.1136/bmj.324.7346.1125 PMC107904

[29] Nabi H, Gueguen A, Chiron M, Lafont S, Zins M, Lagarde E. Awareness of driving while sleepy and road traffic accidents: prospective study in GAZEL cohort. Bmj. 2006;333(7558):75 Epub 2006/06/27. 10.1136/bmj.38863.638194.AE ; PubMed Central PMCID: PMCPmc1489236. 16798754 PMC1489236

[30] Powell NB, Schechtman KB, Riley RW, Guilleminault C, Chiang RP, Weaver EM. Sleepy driver near-misses may predict accident risks. Sleep. 2007;30(3):331–42. Epub 2007/04/12. . 17425230 10.1093/sleep/30.3.331

[31] Golan N, Shahar E, Ravid S, Pillar G. Sleep disorders and daytime sleepiness in children with attention-deficit/hyperactive disorder. Sleep. 2004;27(2):261–6. Epub 2004/05/06. . 15124720 10.1093/sleep/27.2.261

[32] Lecendreux M, Konofal E, Bouvard M, Falissard B, Mouren-Simeoni MC. Sleep and alertness in children with ADHD. J Child Psychol Psychiatry. 2000;41(6):803–12. Epub 2000/10/20. . 11039692

[33] Man KK, Chan EW, Coghill D, Douglas I, Ip P, Leung LP, et al Methylphenidate and the risk of trauma. Pediatrics. 2015;135(1):40–8. Epub 2014/12/17. 10.1542/peds.2014-1738peds.2014-1738 [pii]. . 25511122

[34] Morgenthaler TI, Kapur VK, Brown T, Swick TJ, Alessi C, Aurora RN, et al Practice parameters for the treatment of narcolepsy and other hypersomnias of central origin. Sleep. 2007;30(12):1705–11. Epub 2008/02/06. 18246980 10.1093/sleep/30.12.1705 PMC2276123

[35] Johns MW. A new method for measuring daytime sleepiness: the Epworth sleepiness scale. Sleep. 1991;14(6):540–5. Epub 1991/12/01. . 1798888 10.1093/sleep/14.6.540

[36] Powell NB, Schechtman KB, Riley RW, Li K, Troell R, Guilleminault C. The road to danger: the comparative risks of driving while sleepy. Laryngoscope. 2001;111(5):887–93. Epub 2001/05/19. 10.1097/00005537-200105000-00024 . 11359171

[37] Adler LA, Spencer T, Faraone SV, Kessler RC, Howes MJ, Biederman J, et al Validity of pilot Adult ADHD Self- Report Scale (ASRS) to Rate Adult ADHD symptoms. Ann Clin Psychiatry. 2006;18(3):145–8. Epub 2006/08/23. G5888340H81Q8386 [pii] 10.1080/10401230600801077 . 16923651

[38] Kessler RC, Adler L, Ames M, Demler O, Faraone S, Hiripi E, et al The World Health Organization Adult ADHD Self-Report Scale (ASRS): a short screening scale for use in the general population. Psychol Med. 2005;35(2):245–56. Epub 2005/04/22. . 15841682 10.1017/s0033291704002892

[39] Zigmond AS, Snaith RP. The hospital anxiety and depression scale. Acta Psychiatr Scand. 1983;67(6):361–70. Epub 1983/06/01. . 6880820 10.1111/j.1600-0447.1983.tb09716.x

[40] Rockhill B, Newman B, Weinberg C. Use and misuse of population attributable fractions. Am J Public Health. 1998;88(1):15–9. Epub 1998/05/16. 9584027 10.2105/ajph.88.1.15 PMC1508384

[41] Durmer JS, Dinges DF. Neurocognitive consequences of sleep deprivation. Semin Neurol. 2005;25(1):117–29. Epub 2005/03/31. 10.1055/s-2005-867080 . 15798944

[42] Reynolds AC, Banks S. Total sleep deprivation, chronic sleep restriction and sleep disruption. Prog Brain Res. 2010;185:91–103. Epub 2010/11/16. 10.1016/B978-0-444-53702-7.00006-3B978-0-444-53702-7.00006-3 [pii]. . 21075235

[43] Akerstedt T, Nordin M, Alfredsson L, Westerholm P, Kecklund G. Sleep and sleepiness: impact of entering or leaving shiftwork—a prospective study. Chronobiol Int. 2010;27(5):987–96. Epub 2010/07/20. 10.3109/07420528.2010.489423 . 20636211

[44] Aldrich MS. Automobile accidents in patients with sleep disorders. Sleep. 1989;12(6):487–94. Epub 1989/12/01. . 2595172 10.1093/sleep/12.6.487

[45] Kotterba S, Mueller N, Leidag M, Widdig W, Rasche K, Malin JP, et al Comparison of driving simulator performance and neuropsychological testing in narcolepsy. Clin Neurol Neurosurg. 2004;106(4):275–9. Epub 2004/08/07. 10.1016/j.clineuro.2003.12.003S0303846704000022 [pii]. . 15296999

[46] Sagaspe P, Taillard J, Chaumet G, Guilleminault C, Coste O, Moore N, et al Maintenance of wakefulness test as a predictor of driving performance in patients with untreated obstructive sleep apnea. Sleep. 2007;30(3):327–30. Epub 2007/04/12. . 17425229

[47] Alexandre C, Andermann ML, Scammell TE. Control of arousal by the orexin neurons. Curr Opin Neurobiol. 2013;23(5):752–9. Epub 2013/05/21. 10.1016/j.conb.2013.04.008S0959-4388(13)00097-4 [pii]. 23683477 PMC3783629

[48] Cortese S, Konofal E, Lecendreux M. Alertness and feeding behaviors in ADHD: does the hypocretin/orexin system play a role? Med Hypotheses. 2008;71(5):770–5. Epub 2008/08/06. 10.1016/j.mehy.2008.06.017S0306-9877(08)00286-7 [pii]. . 18678446

[49] Adler LA, Goodman DW, Kollins SH, Weisler RH, Krishnan S, Zhang Y, et al Double-blind, placebo-controlled study of the efficacy and safety of lisdexamfetamine dimesylate in adults with attention-deficit/hyperactivity disorder. J Clin Psychiatry. 2008;69(9):1364–73. Epub 2008/11/18. eJ08M04166 [pii]. . 19012818 10.4088/jcp.v69n0903

[50] Spencer TJ, Adler LA, McGough JJ, Muniz R, Jiang H, Pestreich L. Efficacy and safety of dexmethylphenidate extended-release capsules in adults with attention-deficit/hyperactivity disorder. Biol Psychiatry. 2007;61(12):1380–7. Epub 2006/12/02. S0006-3223(06)00954-1 [pii] 10.1016/j.biopsych.2006.07.032 . 17137560

[51] Biederman J, Fried R, Hammerness P, Surman C, Mehler B, Petty CR, et al The effects of lisdexamfetamine dimesylate on the driving performance of young adults with ADHD: a randomized, double-blind, placebo-controlled study using a validated driving simulator paradigm. J Psychiatr Res. 2012;46(4):484–91. Epub 2012/01/27. 10.1016/j.jpsychires.2012.01.007S0022-3956(12)00008-8 [pii]. . 22277301

[52] Cox DJ, Merkel RL, Kovatchev B, Seward R. Effect of stimulant medication on driving performance of young adults with attention-deficit hyperactivity disorder: a preliminary double-blind placebo controlled trial. J Nerv Ment Dis. 2000;188(4):230–4. Epub 2000/05/02. . 10790000 10.1097/00005053-200004000-00006

[53] Cox DJ, Merkel RL, Penberthy JK, Kovatchev B, Hankin CS. Impact of methylphenidate delivery profiles on driving performance of adolescents with attention-deficit/hyperactivity disorder: a pilot study. J Am Acad Child Adolesc Psychiatry. 2004;43(3):269–75. Epub 2004/04/13. S0890-8567(09)66044-5 [pii] 10.1097/00004583-200403000-00007 . 15076259

[54] Cox DJ, Merkel RL, Moore M, Thorndike F, Muller C, Kovatchev B. Relative benefits of stimulant therapy with OROS methylphenidate versus mixed amphetamine salts extended release in improving the driving performance of adolescent drivers with attention-deficit/hyperactivity disorder. Pediatrics. 2006;118(3):e704–10. Epub 2006/09/05. 118/3/e704 [pii] 10.1542/peds.2005-2947 . 16950962

[55] Cox DJ, Davis M, Mikami AY, Singh H, Merkel RL, Burket R. Long-acting methylphenidate reduces collision rates of young adult drivers with attention-deficit/hyperactivity disorder. J Clin Psychopharmacol. 2012;32(2):225–30. Epub 2012/03/01. 10.1097/JCP.0b013e3182496dc5 . 22367664

[56] Sobanski E, Sabljic D, Alm B, Skopp G, Kettler N, Mattern R, et al Driving-related risks and impact of methylphenidate treatment on driving in adults with attention-deficit/hyperactivity disorder (ADHD). J Neural Transm. 2008;115(2):347–56. Epub 2008/01/18. 10.1007/s00702-007-0834-1 . 18200437

[57] Sobanski E, Sabljic D, Alm B, Baehr C, Dittmann RW, Skopp G, et al A randomized, waiting list-controlled 12-week trial of atomoxetine in adults with ADHD. Pharmacopsychiatry. 2012;45(3):100–7. Epub 2011/12/17. 10.1055/s-0031-1291319 . 22174029

[58] Sobanski E, Sabljic D, Alm B, Dittmann RW, Wehmeier PM, Skopp G, et al Driving performance in adults with ADHD: results from a randomized, waiting list controlled trial with atomoxetine. Eur Psychiatry. 2013;28(6):379–85. Epub 2012/10/16. 10.1016/j.eurpsy.2012.08.001S0924-9338(12)00097-1 [pii]. . 23062837

[59] Verster JC, Bekker EM, de Roos M, Minova A, Eijken EJ, Kooij JJ, et al Methylphenidate significantly improves driving performance of adults with attention-deficit hyperactivity disorder: a randomized crossover trial. J Psychopharmacol. 2008;22(3):230–7. Epub 2008/03/01. 10.1177/02698811070829460269881107082946 [pii]. . 18308788

[60] Ohayon MM, Lemoine P. Daytime consequences of insomnia complaints in the French general population. Encephale. 2004;30(3):222–7. Epub 2004/07/06. MDOI-ENC-6-2004-30-3-0013-7006-101019-ART3 [pii]. . 15235519 10.1016/s0013-7006(04)95433-4

[61] Ohayon MM, Roth T. Prevalence of restless legs syndrome and periodic limb movement disorder in the general population. J Psychosom Res. 2002;53(1):547–54. Epub 2002/07/20. S0022399902004439 [pii]. . 12127170 10.1016/s0022-3999(02)00443-9

[62] Leger D, Poursain B, Neubauer D, Uchiyama M. An international survey of sleeping problems in the general population. Curr Med Res Opin. 2008;24(1):307–17. Epub 2007/12/12. 10.1185/030079907X253771 . 18070379

[63] Leger D, Poursain B. An international survey of insomnia: under-recognition and under-treatment of a polysymptomatic condition. Curr Med Res Opin. 2005;21(11):1785–92. Epub 2005/11/26. 10.1185/030079905X65637 . 16307699

[64] Tison F, Crochard A, Leger D, Bouee S, Lainey E, El Hasnaoui A. Epidemiology of restless legs syndrome in French adults: a nationwide survey: the INSTANT Study. Neurology. 2005;65(2):239–46. Epub 2005/07/27. 65/2/239 [pii] 10.1212/01.wnl.0000168910.48309.4a . 16043793

[65] Ohayon MM, Caulet M, Philip P, Guilleminault C, Priest RG. How sleep and mental disorders are related to complaints of daytime sleepiness. Arch Intern Med. 1997;157(22):2645–52. Epub 1998/04/08. . 9531234

[66] Miano S, Donfrancesco R, Bruni O, Ferri R, Galiffa S, Pagani J, et al NREM sleep instability is reduced in children with attention-deficit/hyperactivity disorder. Sleep. 2006;29(6):797–803. Epub 2006/06/27. . 16796218

[67] Brown TE, McMullen WJ Jr. Attention deficit disorders and sleep/arousal disturbance. Ann N Y Acad Sci. 2001;931:271–86. Epub 2001/07/21. . 11462746 10.1111/j.1749-6632.2001.tb05784.x

[68] Weinberg WA, Brumback RA. Primary disorder of vigilance: a novel explanation of inattentiveness, daydreaming, boredom, restlessness, and sleepiness. J Pediatr. 1990;116(5):720–5. Epub 1990/05/01. . 2329420 10.1016/s0022-3476(05)82654-x

